# From mourning to scientific legacy: commemorating Lister in London and Scotland

**DOI:** 10.1098/rsnr.2013.0038

**Published:** 2013-07-24

**Authors:** Marguerite Wright Dupree

**Affiliations:** Centre for the History of Medicine, University of Glasgow, Lilybank House, Bute Gardens, Glasgow G12 8RT, UK

**Keywords:** Lister, commemoration, Royal Society, museum, Sir Henry Wellcome, centenaries

## Abstract

This paper examines the changing methods, underlying motives, clienteles and controversy surrounding posthumous commemorations of Lord Lister in Britain. The importance of the commemorations for professional identity formation continues throughout the twentieth century, but World War I appears as a turning point. The constituencies commemorating Lister change from broadly international, national and civic with an emphasis on fundraising, to more narrowly professional; the use of religious imagery is notable after the war in the debates in the 1920s; and as his students, so central to the creation and preservation of his image, die, the focus begins to shift from the man and his achievements, ‘the great benefactor of mankind’, to his legacy in the current state of subjects related to his work. The changing nature of the commemorations suggests that although Lister's precise position in the history of surgery is contentious today, his importance as an iconic figure in the history of the medical profession is secure.

The history of commemorations of scientists, including biomedical scientists, has attracted considerable recent interest from historians and is part of a wider growth of concern with commemorative practices and collective memory in other spheres of society.^1^ The collection of studies of past scientific commemorations of individuals, discoveries and institutions brought together and published in *Osiris* in 1999 is a landmark,^2^ which explores the role of cultural context in commemorative practices in science. These studies highlight, for example, the French tradition of celebrating scientists, such as Pasteur, after 1870 as republican substitutes for royalty and saints, and point to the importance of nationalism in the interwar years with celebrations, in 1925, of the centenary of Charcot's birth in attempting to revive the international standing of France after the war. Yet, apart from Patricia Fara's chapter on commemorations of Newton, British commemorations in this collection are noticeable by their absence. However, since then commemoration has been the subject of three presidential addresses to the British Society for the History of Science and a paper comparing commemorations in 1919 surrounding the centenary of James Watt's death and in 1931 for the centenary of Michael Faraday's ‘discovery’ of electromagnetism.^3^

The aim of this paper is to contribute to an understanding of how these events came about and changed their nature and purposes, by focusing on the posthumous commemorations associated with Joseph Lister at different times during the twentieth century. It will both examine public events and take advantage of unpublished sources to go behind them to explore how the activities were organized and to understand the intentions, motivations and relationships of the individuals, institutions, groups and networks involved, and thus how they relate to the broader social, economic, political and cultural contexts in which they took place.

Despite controversy in the 1860s and 1870s surrounding his antiseptic system, Lister was celebrated in his lifetime: created a baronet in 1881, in 1897 he became the first British surgeon to be raised to the peerage and in 1902 one of the 12 original members of the newly founded Order of Merit. After his death, a full page in his funeral programme was insufficient to list the honours and awards from Britain and abroad that had been conferred on him.

As Anne Crowther's paper in this collection suggests,^4^ Lister's reputation and public image by the end of his life was a complex construct, involving both the scientific and moral reputations of the medical profession, the rise of surgery from a craft to a science and the rise of the profession as a whole to a pinnacle of esteem. This image originated in his large Scottish classes, in his experimental approach to surgical practice, in the simplicity and applicability of what students learned from him in lectures, in the moral example he conveyed, and above all in the immense confidence in the future of surgery that he instilled. Although they were not alone, his students, both in London and Scotland, had an important role as creators and guardians of his image and reputation, culminating in his surviving students' contributions to the 1927 centenary celebrations of his birth.^5^

What is less well known is the controversy alongside the commemorations organized in Britain after his death, and the ways in which the commemorations changed in the second half of the twentieth century. What follows will explore the nature of the main commemorations in Britain. Who organized them? What were their aims? What challenges did they face? Which methods did they use? How successful were they in meeting their aims? In what ways did they change over the century?

The Lister Memorial Committees, established in London and Glasgow in the wake of his death in February 1912, had local, national and international aspirations and faced organizational and practical challenges.^6^ The London Committee, although its constituency dwindled after World War I, largely met its objectives and provided the organizational starting point and some of the leadership for the 1927 celebrations of Lister's birth. Yet the constituencies and forms of commemoration in 1927 differed significantly from those planned after his death and from those that followed later in the twentieth century, in 1965 (the centenary of his first successful antiseptic operation) and 1967 (the centenary of his publications in *The Lancet* and *British Medical Journal*). The Glasgow Committee became immersed in the controversy that was to end in the demolition of the Lister Ward at the Glasgow Royal Infirmary in 1924, rather than in its preservation as a museum, as petitions from the major scientific and medical institutions had urged, and in Henry Wellcome's subsequent purchase of its constituent parts. The changing constituencies and forms of commemoration and the controversy reflect the contemporary context and raise issues relevant to commemoration today.

## First commemorations: 1912–24

### Lister Memorial Committees

Lister's funeral, held in Westminster Abbey on 16 February 1912, was attended by the Prime Minister, ambassadors, representatives of royalty and the scientific and medical elite from around the world, as well as more humble practitioners and nurses. The attendance underlined his international stature. A ‘beautiful wreath of orchids and lilies sent by the German Emperor’ was ‘carried before the coffin as the procession entered the Abbey’.^7^ The music, the lack of an address, and three of the pallbearers ‘stirring the procession to unwonted colour by the brightness of the gowns they wore’ were identical to features of the funeral of Lord Kelvin in the Abbey five years previously, and *The Times* described the ceremony as ‘one of the most impressive ever held in the Abbey; one in which every one present was a sincere mourner’.^8^

In the wake of his death, Lister Memorial Committees were established, first in Glasgow and then in London. They faced organizational and practical problems. A group in Glasgow acted quickly: within 10 days of the funeral, the Lord Provost called a meeting that unanimously resolved that Glasgow should commemorate the achievement of the great pioneer of modern medicine whose discoveries had done ‘so much for the alleviation of human suffering’.^9^ Two weeks later ‘a representative meeting of the citizens of Glasgow’ proposed that the memorial should be both local and international.^10^ Given Lister's connections with the Royal Infirmary, they proposed ‘a monument of artistic design in the neighbourhood’ of the Infirmary, and three more ambitious proposals with an ‘international character’, including the establishment of a Lister Award for contributions to surgery ‘in any part of the world’, a Lister Research Fund for promoting fellowships and studentships, and the conversion of the Lister Ward in the Royal Infirmary into a museum.^11^ They faced the question of how to organize a commemoration that had local and international dimensions, involving a range of institutions.

In London, Fellows of the Royal Society and the Royal College of Surgeons of England discussed the question of a memorial to Lister immediately after his death, but they were slow off the mark in comparison with Glasgow, and it was not until May that a Provisional Committee, consisting of seven representatives of the Royal Society, the Royal College of Surgeons of England and the Royal Society of Medicine, including Rickman Godlee, Lister's nephew and President of the Royal College of Surgeons of England, and William Watson Cheyne, met under the chairmanship of the President of the Royal Society, with Sir John Rose Bradford of the Royal Society acting as secretary.^12^

The Provisional Committee realized that no single institution had the prestige and structure for decision-making that could coordinate the wide range of individuals and institutions interested in Lister's commemoration, produce agreement on a suitable memorial and organize the collection of subscriptions. So, instead, it took steps to create a new entity, the Lister Memorial Committee, made up of a large General Committee, which would appoint a smaller Executive Committee to prepare, raise funds for and carry out a scheme. To maximize support and resources it invited the widest possible range of prominent institutions and individuals to form the General Committee, including approximately 80 institutions, and a vast array of individuals, ‘not only men distinguished in science and surgery, but also men of eminence in public life and in various branches of knowledge’, including Ambassadors and Ministers to the English Court, High Commissioners and Crown Agents, the Archbishop of Canterbury, the Prime Minister, the Master of the Rolls, the Speaker, the Viceroy of India, the Lord Provosts of Edinburgh and Glasgow and the Lord Mayor, HRH Prince Arthur of Connaught and HRH Prince Louis of Battenberg.^13^

At its initial meetings in May, the Provisional Committee in London also had to work out its relationship with the existing Lister Memorial Committee in Glasgow. This highlights the utility of the General Committee structure envisaged in London. The Glasgow Committee would be well represented on the General and Executive Committees in London, and conceded that any international memorial would be initiated in London, and the form of commemoration would be decided by these committees, whose first meeting would be in July.

In addition, a Glasgow delegation and the London Committee discussed the knotty question of raising subscriptions for local purposes in Glasgow and agreed that appeals could be made in Glasgow for subscriptions to be devoted to either a local or an international memorial, or both.^14^

The large number of invitations sent out by the Provisional Committee in London resulted in the attendance of 37 people at the initial meeting of the General Committee in July 1912 in the rooms of the Royal Society in Burlington House.^15^ The meeting approved the appointments of Watson Cheyne as treasurer, Sir John Rose Bradford as secretary, and 27 members of the Executive Committee, which still included prominent figures such as the Archbishop of Canterbury, the Lord Chancellor, Master of the Rolls, Viscount Iveagh, the Lord Provosts of Edinburgh and Glasgow, and the Lord Mayor of London, as well as the leaders of medical institutions, for example the presidents of the General Medical Council, the Royal College of Surgeons of England and the Royal College of Physicians, London.

Regarding the form of the memorial, the Executive Committee made four proposals, starting with a memorial in Westminster Abbey and monument in London and including a major prize for contributions to surgery or its science, and the preservation of the Lister Ward in Glasgow as a museum:

(i) a memorial in Westminster Abbey to take the form of a medallion or tablet, as space was limited;
(ii) a monument in a public place in London;
(iii) an International Lister Memorial Fund for the advancement of surgery, from which either grants in aid of research bearing on surgery, or awards in recognition of distinguished contributions to surgical science, should be made irrespective of nationality, somewhat on the lines of the Nobel Prize;
(iv) that in any appeal issued attention be called to the suggestion that has been made that one of the wards of the Royal Infirmary, Glasgow, should be preserved as a museum in which objects of interest associated with Lord Lister and his discoveries might be exhibited.^16^

### Fundraising strategy

Also in July 1912, plans for fundraising began in earnest and will sound familiar to fundraisers today. The first step was a meeting at the Mansion House hosted by the Lord Mayor of London to launch the appeal. A date in October was arranged and a list of speakers was drawn up, headed by the Prime Minister. In the end he was unable to attend, but the Lord Chancellor and others spoke.^17^

Before the Mansion House meeting the Executive Committee agreed the content of the appeal for subscriptions. The issue of whether the Lister Ward was international or local was again negotiated and, to the dismay of the Glasgow representatives, dropped from the body of the main appeal. Instead, a notice was inserted as a leaflet in the appeal to preserve

one of the wards of the Royal Infirmary … where Lord Lister's antiseptic methods were first put into practice … as a museum in which objects of interest associated with him and his discoveries might be exhibited. … the Directors of the Infirmary have given their sanction to the scheme. Objects, personal or otherwise, associated with Lister's life and work, are earnestly desired, and will be gladly received by the Superintendent, Royal Infirmary, Glasgow.^18^

The Mansion House meeting raised donations of just over £800, providing an initial list of donors associated with the appeal. The appeal was widely targeted in Britain and Ireland, starting with heads of universities, presidents of the medical societies and of the branches of the British Medical Association (BMA), the mayors of London boroughs and large provincial towns, City livery companies, scientific societies, the principal hospitals, and a selected list of members of the medical profession and other individuals likely to be interested. London bankers, members of Lloyds and the Stock Exchange were included, and advertisements were inserted in newspapers.^19^

### Fundraising results

**Figure 1.**
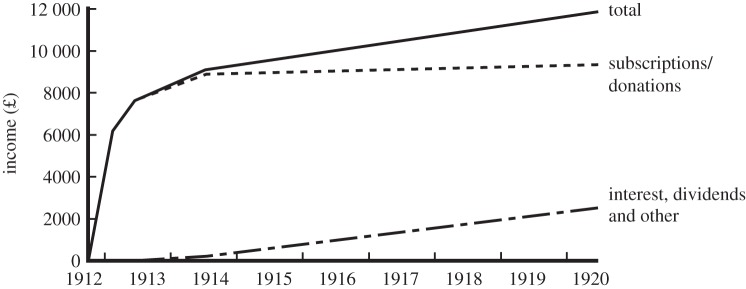
Cumulative income for the Lister Memorial Fund, 1912–20. (Data source: Lister Memorial Committee, Royal Society Archives, CMB/13.)

Most donations (figure 1), more than £6000, came in the first six months between the Mansion House meeting in October 1912 and the end of February 1913.

A second round of newspaper advertising went out after Easter 1913. At this stage the colonies and foreign countries were approached, via an appeal sent to 277 presidents of the universities and societies that had made Lord Lister a member or awarded him an honorary degree.^20^ Over the next four months, a further £1500 was received, including £323 from the appeal abroad, bringing the total to about £7600 by June 1913.^21^ During the next year donations flattened out, with £1200 being added by July 1914. By then, a total of 17 500 appeals had been sent by post to the medical profession and the general public in the UK and 300 letters to institutions in the colonies and foreign countries. Donations amounted to £8888.^22^

**Figure 2.**
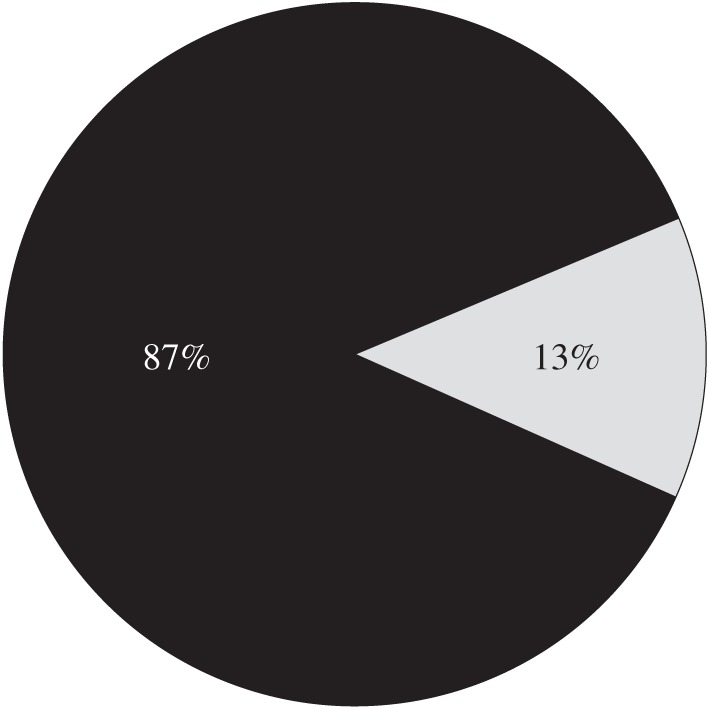
Geographic origins of subscription income for the Lister Memorial Fund. (Data source: Lister Memorial Committee, Royal Society Archives, CMB/13.)

In the end, the bulk of the donations (87%) came from Great Britain and Ireland, with the rest (about £1000) from the colonies and foreign countries (figure 2). Although donations by 1914 amounted to approximately £700 000 at today's prices, the Committee was disappointed that the contributions were not enough to endow adequately a Surgical Research Fund.^23^ Lister's universal acclaim had practical limits.

### Implementation

**Figure 3.**
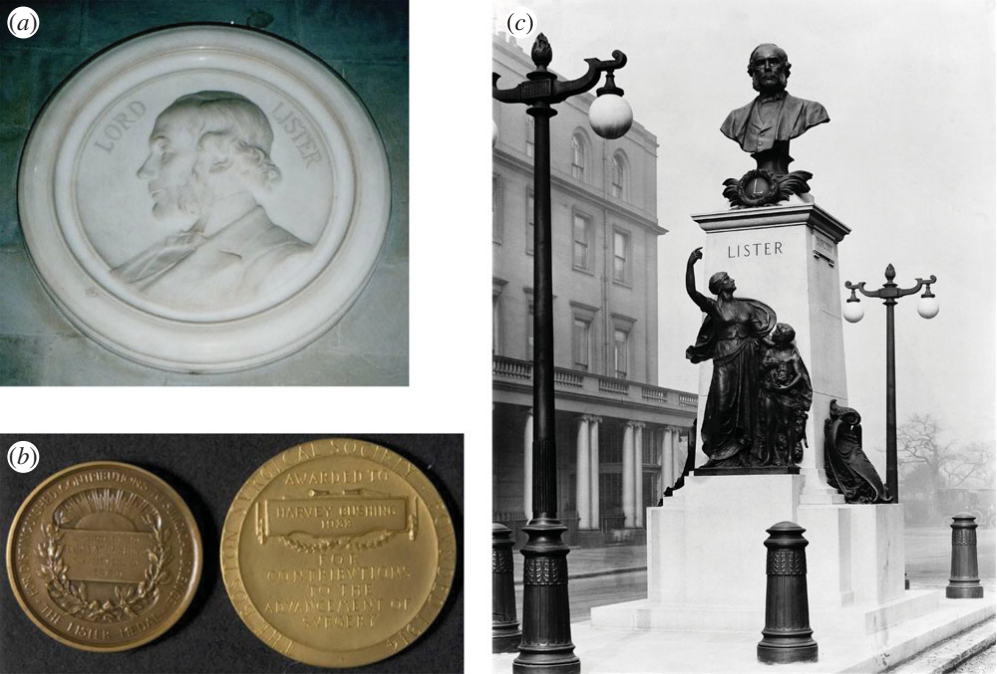
Implementation of the memorial plans. (*a*) Marble medallion in Westminster Abbey, unveiled 1915. (Source: Victorian Web (http://www.victorianweb.org/sculpture/brock/39.html); reproduced with permission of the photographer, John Sankey.) (*b*) Lister Medal and Lecture, initiated 1924. (Source: Yale University, Harvey Cushing/John Hay Whitney Medical Library; reproduced with permission.) (Online version in colour.) (*c*) Statue in Portland Place, unveiled 1924 (Source: Wellcome Library, London; reproduced with permission.)

However, by June 1913 the Lister Memorial Fund was large enough to begin to implement the memorial plans. The first priority was the plaque in Westminster Abbey (figure 3*a*). A site in the Abbey near Darwin's burial place was agreed, and Sir Thomas Brock, well known for the statue of Prince Albert in the Albert Memorial and for the statue of Queen Victoria in the Victoria Memorial in front of Buckingham Palace, accepted and completed the commission for £700 in 1915.^24^

No further action took place until 1920, but this also meant that there was little expenditure and the bulk of the donations were invested.^25^ So, despite little in the way of further donations, the Fund did well out of the war, adding £2300 from investments, or 25% to the £9332 received from subscriptions. Yet with postwar inflation it lost roughly half its prewar value.

After the war the Executive Committee picked up where it had left off.^26^ There was continuity in the individuals most closely involved, including Bradford, Cheyne and Godlee. It moved quickly to establish an International Lister Memorial Fund for the advancement of surgery, which provided a bronze medal (figure 3*b*), awarded every three years, irrespective of nationality, in recognition of distinguished contributions to surgical science, and required the recipient to give an address in London under the auspices of the Royal College of Surgeons of England. The award would be made by a committee with seven representatives of the Royal Society, the Royal Colleges of Surgeons and the universities of Edinburgh and Glasgow. Any surplus income of the Fund would be either devoted to furthering surgical science by means of grants or re-invested. A challenge for the existing Lister Memorial Committee was to ensure that this part of the Memorial would be on a permanent basis, because the Committee was not permanent. As a result, the Royal College of Surgeons of England agreed to become the Trustees and administer the Fund.^27^

The Executive Committee also moved quickly to erect a monument in a public place. It arranged a site in Portland Place and invited Sir George Frampton (1860–1928), best known for his statue of Peter Pan in Kensington Gardens, to undertake the commission for 4000 guineas. The Committee's members wanted Frampton so much that when he initially replied that he would not be able to begin work within the next four years, the Committee, in its only recorded vote, decided to commission him anyway. To their dismay, in the end, Frampton declined the invitation.^28^

After this rebuff, the Committee turned again to Sir Thomas Brock, who accepted. By June 1922 Brock had completed the bust and sent it to the foundry; the large female figure was nearly complete, and only the figure of the boy remained. Then near disaster struck: in August Sir Thomas Brock died. Fortunately the work was far enough along that Brock's assistant could complete the memorial (figure 3*c*), although the Committee required assessment by an outside expert before allowing him to proceed.^29^

The King, the Prince of Wales and Lord Balfour turned down invitations to unveil the statue. In the end, the President of the Royal Society performed the ceremony in 1924, before ‘a small but representative gathering’. According to *The Times* the ceremony ‘was brief and quiet but lost nothing in effectiveness for that’. Also in 1924 the unexpended balance of the Fund was handed to the Royal College of Surgeons as arranged, and the Lister Memorial Committee ended.^30^

The Lister Memorial Committee had succeeded in, but not exceeded, its aims. It left the memorial in Westminster Abbey, the statue in Portland Place in London, and the international Lister Medal for surgery, and the Lecture and fund administered by the Royal College of Surgeons of England. Having started with a large constituency of prominent men, ‘not only distinguished in science and surgery, but also … of eminence in public life and in various branches of knowledge, both at home and abroad’,^31^ there was only a handful remaining at the end.

It is not clear whether sufficient contributions, donations and bequests would have been forthcoming to establish a Surgical Research Fund as the Lister Memorial Sub-Committee hoped at its last meeting on 24 July 1914, but the outbreak of World War I days later made failure a certainty. Mourning and memorials became a major preoccupation during and after the war throughout the UK and combatant countries, as financial resources and emotional energy were directed to collective commemoration.^32^ Moreover, the sharp downturn in the UK economy hindered fundraising drives immediately after the war, as those attempting to commemorate the centenary of James Watt's birth found in 1919.^33^

## Later commemorations: 1927–67

### The Lister Centenary 1927

However, less than two years after the Lister Memorial Committee transferred the final tranche of funds to the Royal College of Surgeons, that committee became the model for the organization to arrange the celebrations in London for the centenary of Lister's birth, and some of the same individuals reappear. Again the Royal Society took the lead. At the end of April 1926 the Society's President chaired a meeting of delegates unanimously in favour of a celebration to commemorate the centenary of the birth of Lord Lister.^34^ The meeting appointed an Executive Committee to ‘formulate proposals, but without plenary powers’. Again, Sir John Rose Bradford served as its secretary.

This meeting of delegates, however, differed from the large General Committee with plenary powers of 1912. Gone were the Archbishop of Canterbury and Master of the Rolls, the Ambassadors, the Speaker and the Prime Minister; gone was the combination of institutions and individuals. Instead there were delegates from 51 leading medical institutions in the country: the Royal Colleges, the Royal Society, the Society of Apothecaries, medical societies, 11 major London hospitals and the Lister Institute, the Glasgow Royal and Western Infirmaries, the Edinburgh Royal Infirmary, and 18 universities and colleges with medical schools. Watson Cheyne was the only individual invited in his own capacity. This was to be a celebration by the medical profession.

As can be seen from the programme (table 1), the centenary celebrations in London in 1927 spread over three days centred on 5 April, Lister's birthday. There are several points to note about the programme. First, it included national recognition of the importance of surgery, surgeons and the medical profession more generally in the form of the reception by the King at Buckingham Palace to open the celebrations, and the next day a reception by the Prime Minister at BMA House. The latter included appreciations by a French delegate and a German delegate, emphasizing the international dimension of the celebration. Second, the afternoon and evening of the first day were entirely taken up with reminiscences of Lister by his surviving students, dressers and assistants. They stressed his achievements and his exemplary personal characteristics. Third, on the afternoon of the third day there were three addresses about Lister in relation to current work in fields to which he contributed, foreshadowing the nature of future Lister commemorations, which emphasized his legacy. Finally, the exhibition of the Lister Collection at the Wellcome Historical Medical Museum was on the programme, although the reception that the museum held for 500 delegates on 6 April was not allowed to be listed.^35^

**Table 1.** Programme of the Lister Centenary celebrations in London, 1927.

	**Monday, April 4th**
11.00 a.m.	Reception of delegates at Buckingham Palace by HM the King
3.00 p.m.	King's College Hospital. Addresses by Watson Cheyne, Bt, and eight others on ‘Lister's Personality’
8.30 p.m.	Reception at Royal Society of Medicine. Address by Sir St Clair Thomson: The Centenary of Lister—Recollections by One of his House-Surgeons
	**Tuesday, April 5th**
11.30 a.m.	Reception of official delegates and members of the Lister family by the Prime Minister at the BMA House
4.00 p.m.	Conversazione at the Royal College of Surgeons of England, Lincoln's Inn Fields
7.30 p.m.	Dinner by Merchant Taylors Company at 30 Threadneedle St.
	**Wednesday, April 6th**
11.15 a.m.	Memorial Service at Westminster Abbey
3.00 p.m.	Address at the house of the Royal Society of Medicine by (a) Sir Charles Sherrington on Lister as a Physiologist; (b) Professor W. Bulloch on Lister as a Pathologist; and (c) Sir Berkeley Moynihan on Lister as a Surgeon
9.00 p.m.	Conversazione at the rooms of the Royal Society, Burlington House

The Lister Collection in the Wellcome Historical Medical Museum, 54A Wigmore St., will be open from 9.30 a.m. to 6 p.m. on and after Monday, April 4th.

The London celebration was not the only Lister Centenary celebration in 1927.^36^ Among others were one in Glasgow on 1 April, one in Carlisle on 5 April to mark the town's connection with Lister's use of carbolic, and the BMA's annual meeting in Edinburgh in July, which was a celebration of the centenary, including an exhibition of 298 ‘Lister Relics’ and a volume containing reminiscences of his students and assessments of developments in fields related to his work.^37^

The Lister Centenary celebrations in London in 1927 were a celebration of his achievements and personal attributes. Yet there was also a changing constituency for the commemorative activity from that immediately after Lister's death, a new attempt to assess his legacy for surgery and related fields, and in 1927 no attempt to raise money.

### Lister centenaries: Glasgow 1965 and London 1967

Since 1927, despite the deaths of his remaining former students, the medical profession in particular continued to recognize and celebrate Lister centenaries and use them to assess developments in areas of medicine and science related to Lister's work.^38^ Centenary commemorations in Glasgow in 1965 marked his development and application in 1865 of antiseptic surgical techniques, and celebrations in London in 1967 commemorated the centenary of their first publication in *The Lancet* in 1867. Both events were detached from consideration of Lister as a person, although not for the reasons of personal scandal that lay behind the celebration of the 40th anniversary of Koch's ‘discovery’ of the tubercle bacillus in 1922.^39^ The commemorations no longer emphasized Lister as a ‘moral exemplar’ but instead focused in 1965 on his legacy for current issues in wound management and in 1967 on safe surgery.

**Figure 4.**
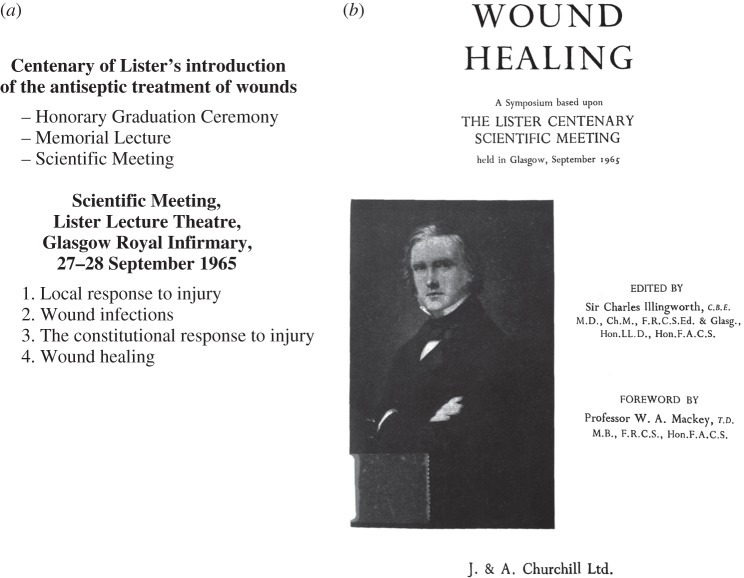
Lister Centenary celebrations, Glasgow, 1965. (*a*) Programme of the meeting. (*b*) Title page of the symposium proceedings.

In September 1965, to mark the centenary of Lister's introduction of the antiseptic treatment of wounds in the Glasgow Royal Infirmary, two postage stamps were issued giving national recognition to his achievement, and the University of Glasgow and Glasgow medical institutions held a centenary celebration, including an honorary graduation ceremony, a memorial lecture and a scientific meeting (figure 4*a*).^40^ Under the general title ‘Progress since Lister’, the scientific meeting comprised a series of papers on wounds and related topics and was held over two days in the Lister Theatre in Glasgow Royal Infirmary. The 31 participants came from medical schools and institutes around the world, and the papers were published together in a single edited volume (figure 4*b*). The symposium associated Glasgow and the university with innovation and progress, and was seen as a ‘fitting part of the commemoration of the work which [Lister] began a hundred years ago and which remains relevant, viable and productive to-day … the contributions … [being] exciting and stimulating, forward-looking rather than retrospective’.^41^

**Figure 5.**
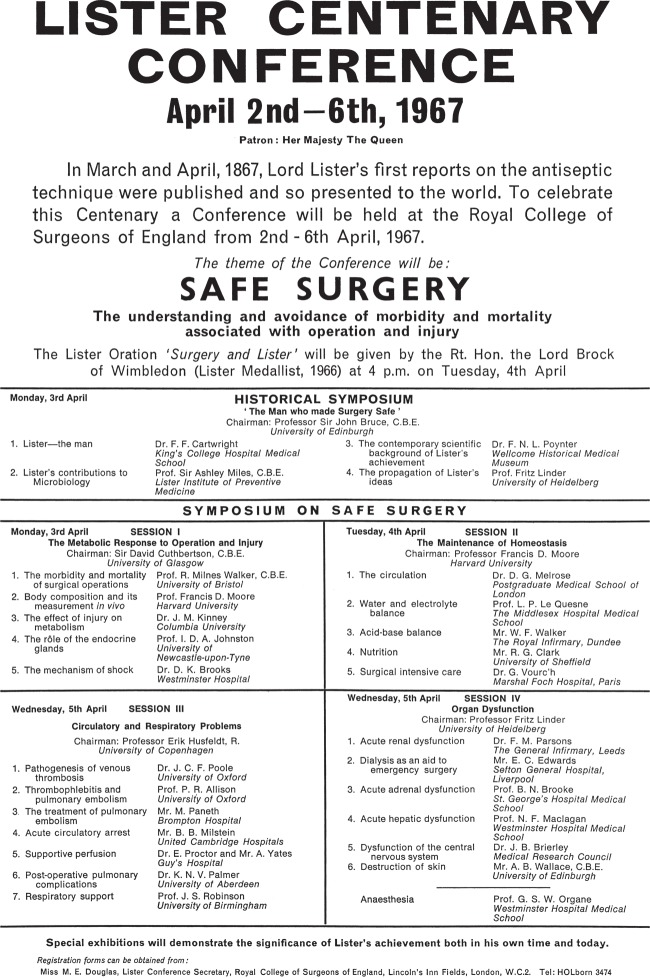
Lister Centenary celebrations, London, 1967.

In 1967 the Royal College of Surgeons of England took the initiative and organized a major conference in London to commemorate the centenary of the publication of Lister's first papers on antiseptic surgery.^42^ Close to the dates of their publication in March and April, the conference coincided with Lister's birthday, 5 April. The Queen agreed to be the patron of the conference and sent a message. The conference theme was ‘Safe surgery’, and although most of the programme was devoted to a scientific meeting, there was also a ‘historical symposium’ (figure 5).

As an adjunct to the conference on 6 April, King's College Hospital arranged a meeting that used the celebration of Lister, among other things, as a way to showcase recent developments. Because Lister was a pioneer of implants in surgery, Michael Debakey gave a talk to an overflow audience about his work on materials for heart valve replacements, including a film of them in operation.^43^ Similarly, Edinburgh held a ‘scientific symposium on antibiotics’.^44^

Thus, since his death, commemorations of Lister have taken a wide variety of forms. According to the *Glasgow Herald*, the variety of commemorative options was one of the attractions of the proposals put forward by the Glasgow Lister Memorial Committee in 1912. Yet one of the Glasgow Committee's proposals, namely to preserve a Lister Ward in the Glasgow Royal Infirmary and create a museum, caused a major public controversy.

## Controversy: the Lister Ward

As mentioned above, in October 1912 the establishment of a Lister Museum in the Lister Ward of the Royal Infirmary failed to retain its designation as an ‘international’ memorial that would benefit from the ‘international funds’ raised by the Lister Memorial Committee in London. Nevertheless, by the time it launched its separate appeal at the end of December 1912, the Glasgow Committee was proud of having been first in the field and of the ‘hearty co-operation’ with the London Memorial Committee.^45^ When a delegation from Edinburgh suggested to the Glasgow Committee that it join in promoting an appeal for a Scottish national memorial to Lister in the form of research laboratories, the Glasgow Committee persuaded them that this was already met by the aims of the London Committee, the strong Scottish representation on it, and the arrangements for shared fundraising, for both international and local forms of memorial.^46^

**Figure 6.**
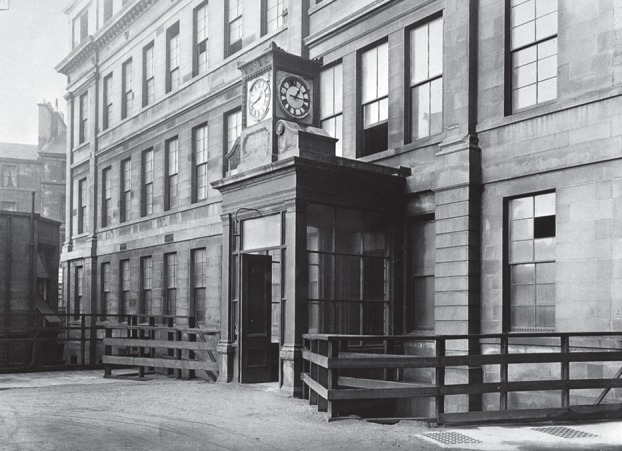
Lister Ward exterior (Male Ward 24, first floor, left of entrance) before demolition, *ca*. 1924. (Photo by T. & R. Annan & Sons; Wellcome Library, London; reproduced with permission.)

With the proposal to preserve a Lister Ward (figure 6) and create a museum in it providing the main local focus for the appeal, one of the Glasgow Committee's first steps in March 1912 was to obtain the approval of the Managers of the Glasgow Royal Infirmary. The approval did not bind future Managers, and there was some opposition: the motion passed by 12 votes to 8. The supporters, however, took the view that, as time passed, interest and support would increase, making any reversal unlikely.^47^

In 1912 the Royal Infirmary was coming to the end of a controversial, costly complete rebuilding on the same site, rather than move to a more spacious suburban location. Beginning in 1905, the project involved the demolition of the original eighteenth-century building designed by Robert Adam and the surgical block, opened in 1864, where Lister had worked. In 1912 the surgical block was still in use and not due for demolition until 1914.^48^ Plans proceeded smoothly. The Glasgow Lister Memorial Committee raised about £2600 from its local appeal and acquired relics for the museum, after the Mansion House meeting and the leaflet inserted in the London Committee's appeal in 1912.^49^

Suddenly in April 1914 the Managers voted to revoke the 1912 approval for the retention of the Lister Ward by 13 votes to 8. The grounds for removal included several concerns: (i) it was a septic menace to the neighbouring wards in the new building; (ii) it was an ‘obstruction to light and air’; (iii) it occupied space needed for other purposes; and (iv) it was out of keeping with the new building, and interfered with a proper view of these buildings from the public thoroughfares.^50^ Correspondence appeared in local and national periodicals, overwhelmingly against the action of the Managers, and supporters refuted the reasons given, point by point.^51^

The University of Glasgow offered to rebuild, on its campus, the ward and operating theatre from the original materials, complicating the issue for supporters of retention. The Managers voted again to demolish the ward, but to offer the materials to the University. Acrimony crept in when one of the Managers referred to the structure as ‘merely so much stone and lime both worthless and injurious as an adjunct to the Infirmary’; the statement angered those ‘who regarded this workshop of Lister's with a feeling of respect which amounted almost to reverence’.^52^

With the outbreak of war, discussion ended, and the ward was used for injured soldiers. At the end of the war the Managers faced a severe financial crisis and decided against immediate demolition of the ward, because the cost had trebled since 1914 as a result of postwar inflation.^53^

In 1921 Glasgow University withdrew its ‘offer to accept the materials of the Lister Ward for re-erection at Gilmorehill … because the site was to be put to another use’.^54^ In view of this withdrawal and pressure from the Glasgow Lister Memorial Committee, the Managers held a special meeting in October 1921, and again considered whether it was in the interests of the Infirmary to retain the ward.^55^ The ensuing discussion highlighted the opposing views: two Managers advocated the retention of the ward as the birthplace of antiseptic surgery, but the new chairman advocated removal, saying ‘the want of free space applied as much, if not more, to the Infirmary as to the University’; and another Manager further articulated the case for demolition, arguing ‘that the whole of the reconstructed Infirmary was the best Memorial to Lord Lister’, as its design was largely the outcome of his discovery. The Managers were charged with the preservation of the best interests of the Infirmary, and the permanent retention of the ward as a Lister Museum, however admirable in sentiment, was inconsonant with the true purposes of the institution.^56^

The Managers again voted to remove the ward. The Glasgow Lister Committee turned its efforts to commissioning the statue of Lister as a memorial, unveiled in Kelvingrove Park (rather than the original site adjacent to the Infirmary) in September 1924 (see the frontispiece to this issue of *Notes & Records*), but did not end its efforts to save the ward. At the start of 1922 it launched a new campaign. In July 1922 the BMA held its annual meeting in Glasgow. There were tours of the Lister Ward, vigorous support for its retention, and dismay at the situation in which ‘a shrine so unique in itself and which by virtue of its association belongs not to Glasgow only but to the civilized world should be wantonly destroyed’.^57^

The supporters had to make the case for the value of the retention of the ward as a museum on its original site, as opposed to other means of commemoration, such as a monument or a reconstruction. To do this the supporters drew heavily on religious imagery and analogy. The Glasgow architect James Morris, for example, argued that the ward was more than its building materials: ‘the disintegration of the material body carries with it severance from the soul, in the building, just as in the spiritual world’.^58^

Support from the employee contributors whose funds were important to the Infirmary reinforced the Managers' position. So, with time running out, in September 1923 the Memorial Committee launched an even wider appeal to increase the pressure on the Managers to reverse their decision. They sent letters and printed circulars to prominent individuals and medical institutions at home and abroad. An editorial in *The Times* headed ‘Vandalism in Glasgow’ attacked the attitude of the Infirmary's Managers.^59^

The response to these appeals exceeded the Committee's expectations. Petitions to the Managers to retain the ward started rolling in from the French Academy of Medicine, the International Society of Surgeons of Belgium and other continental medical and surgical bodies. More than 100 ‘of the most distinguished citizens of Glasgow’ signed a petition in support of retention of the ward, including the Moderator of the General Assembly of the Church of Scotland, the Roman Catholic Archbishop of Glasgow, the President of the Royal Society of Edinburgh, two former Lord Provosts of Glasgow, and Members of Parliament. Funds were offered to the Managers, so cost was not a decisive factor. Most support came from the medical profession and especially from surgeons at home and abroad.^60^

In December 1923, despite the petitions and the offers of funding, the Managers voted again decisively to demolish the Lister Ward.^61^ Yet even this vote and the start of demolition on 21 January 1924 did not end the campaign to preserve the ward. A new appeal suggested that ‘if intervention is quick, the evil may even yet be averted’.^62^ In the House of Commons, MPs argued that the ward ‘should be regarded as a national monument to a great benefactor of mankind’ and asked the Secretary of State for Scotland to intervene to prevent demolition; the Secretary of State replied he did not have the authority.^63^ The protests were to no avail. By the end of May ‘the last stone of the famous Lister Ward was cast down’.^64^

Thus, the Glasgow Lister Memorial Committee succeeded in its aim of raising funds for a local monument to Lister to commemorate his link with the city and carried it out in the form of the statue in Kelvingrove Park, although not in its expected location opposite the Royal Infirmary. It also succeeded in collecting Lister ‘relics’ for a museum. However, it failed spectacularly to commemorate Lister through the preservation of the Lister Ward at the Royal Infirmary to house a museum. Why, despite worldwide appeals presented to the Managers, was it removed?

The final decision in December 1923 was in the hands of the 36 Managers of the Royal Infirmary, under the chairmanship of a businessman who had been a Manager for nearly 25 years. The controversial rebuilding of the hospital, the resulting financial crisis, exacerbated by the war and postwar inflation, the role of the University of Glasgow's offer to rebuild the ward on its campus, and the composition of the Board of Managers are important in understanding the decisions.^65^

**Figure 7.**
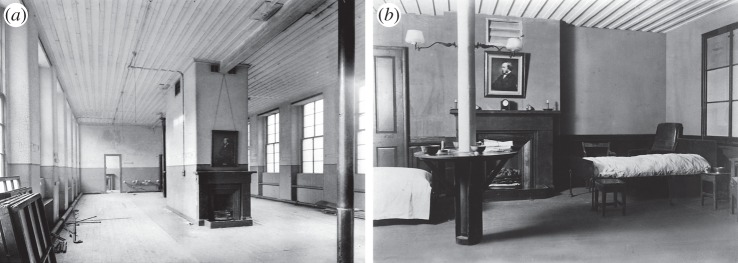
Lister Ward compared with its re-creation in the Wellcome Historical Medical Museum. (*a*) Interior of Male Surgical Ward 24 before demolition, *ca.* 1924. (Photo by T. & R. Annan & Sons; Wellcome Library, London; reproduced with permission.) (*b*) Reconstruction of Lister Ward in the Wellcome Historical Medical Museum, *ca.* 1927. (Wellcome Library, London; reproduced with permission.)

But the fundamental divide was between irreconcilable views of the relative value of different forms of commemoration. To the preservationists, the ward was not just ‘rocks and lime’. It was the building as part of the Royal Infirmary and the space within it where Lister had worked that were crucial (figure 7), both for understanding and for commemorating him and his achievement. Hence there were references to ‘reverence’, ‘sentiment’, a ‘shrine’, a ‘place of inspiration and learning’. Like a chapel, they argued, it was an appropriate part of a modern hospital, and it offered a unique opportunity for the Infirmary and the city.

For those who favoured demolition, preservation of the ward as a museum was outside their concept of the purpose of the hospital, namely the healing of the sick. Because it was old, it was also not an appropriate part of a new, modern hospital. There were aesthetic grounds, too, for opposition: it was not part of the architect's plan for the new hospital. And on practical grounds of efficiency, with ground space in short supply, the opportunity cost was too high.

If the assessment of the value of the ward as a building integral to the Royal Infirmary, namely as a ‘shrine’, was not sufficient for its preservation *in situ*, the value of its component parts as ‘relics’ was soon clear.

## Collection

In January 1924, three days before the demolition started, C. J. S. Thompson, the curator of the Wellcome Historical Medical Museum (WHMM) began negotiations with the demolition company that now owned the surviving furniture, fittings and eventually stones and timbers.^66^

Sir Henry Wellcome had established the WHMM in London before the war.^67^ He amassed a vast collection of objects intending to illustrate the development of the many branches of medicine and the healing arts from their beginnings to the present, and to conserve the relics of key discoverers in those lines of development as a permanent tribute. For this project he considered ‘the work of Lord Lister and the surroundings in which he worked to be of first importance’.^68^ The collection of Lister relics had a key early role in the creation of the museum as a ‘well-oiled collection machine’ to fulfil Wellcome's aim of creating a collection encompassing the complete history of medicine.^69^

Seeing the Glasgow material in January, Wellcome's curator decided not only to preserve objects, but also to reconstruct a corner of the ward in the museum ‘to form a permanent record’ of Lister's work (figure 7). He arranged for photographs, and by April 1924 he had both purchased the bath, ventilator, round table, door, mantelpiece and window sash from the demolition firm, and had persuaded the Infirmary's Managers to give the museum a few articles of the period, including two beds, a fracture board, a ward armchair, two ward night stools, two small bedside stools, a rough old dresser and a gas bracket, although he admitted that ‘none of these articles can be connected with the Ward in Lister's time’.^70^

The clear importance of Lister to the museum stimulated further collection, and the staff adopted a policy of following up ‘all important relics’ by returning to Glasgow where material was still available from the contractors. This time the new curator, Louis Malcolm, and his colleague Johnston Saint proudly reported to Wellcome that they had acquired ‘window sashes with original glass, 160 thirteen-foot-long joists, mahogany rails and irons, a 30-foot long main beam, a round table, three basins, three ventilators, the two leaf-doors of the ward, etc.—all at house-breakers’ prices, £74.15.5d'.^71^ This success led the curators to formulate a general ‘system of following up all material which may be available, belonging to eminent men’.^72^

In the case of Lister, the follow-up also included much contact with the Lister family and led to the acquisition of material from the Lister Institute. In addition, the museum compiled a list of Lister's former dressers, clerks and house surgeons and wrote to each asking for letters and items related to Lister. By early June 1926 the curator told Henry Wellcome ‘we have practically the whole of Lister's material in our possession, either on permanent loan or by presentation’.^73^

The timing could not have been better. Henry Dale, from the newly formed committee to celebrate the Lister Centenary in London in April 1927, visited the museum. He was impressed. He told the curator that ‘there was a proposal that a temporary building should be put up in the court at Burlington House as a Lister Museum for the centenary, but he said that the RCS [Royal College of Surgeons of England] was not going to allow their material out of their building.’ The Wellcome Curator replied immediately he was certain that Henry Wellcome would not allow his Lister material to be moved.^74^

In the end, as we have seen, the WHMM did house the official Exhibition of the London Lister Centenary listed on the programme, and held a reception in the museum for delegates on the final day. The museum used the exhibition to extend its collection, in competition with other collectors. Having acquired objects on loan for the exhibition, the museum offered at its conclusion to provide a permanent home.^75^ In addition, Wellcome held to his policy of not lending objects from his collection. Instead, he had duplicates made for the Lister Centenary exhibitions in Edinburgh in July and in the USA, thus not missing the opportunity to raise the profile of the collection and enhance its status, particularly among his intended audience: the medical profession.

## Conclusion

What can we learn from this brief survey of Lister commemorations in Britain since his death?

First, Lister's universal acclaim had its limits. The London Lister Memorial Committee developed an effective representative organization based in the Royal Society, and it raised enough funds for its specific aims but not enough for the large surgical research fund that had been envisaged. At the same time, the Glasgow Lister Memorial Committee represented Glasgow nationally and was successful in fundraising locally, yet it failed in its aim to preserve the Lister Ward.

Second, although professional identity formation for surgeons is a continuing theme throughout the twentieth century, World War I marked a change in the constituencies for commemorating Lister from broadly international, national and local, to more narrowly professional. In addition, in the 1920s religious imagery characterized the memorialists in the postwar debate about commemoration, emerging strongly in the words used, for example ‘relics’, ‘body’ and ‘soul’, and especially ‘shrine’.

Third, controversy over the Lister Ward emphasizes that Lister's is a living heritage that is inherently controversial. It raises issues of what is the best form of surgical commemoration. Is it the continuing work of the hospital itself, as the Managers argued? Or is it a museum? However, even if it is a museum, the vision of the supporters of the retention of the Lister Ward, although similar in many ways, was not what ended up as the Lister Ward reconstructed in the WHMM, and it raises wider issues surrounding the preservation of historic buildings.

Finally, while the medals and orations continue,^76^ the forms of commemoration since Lister's death have tended to shift from plaques and sculptures in public places to gatherings to celebrate achievements and discuss the current state of subjects related to Lister's work. In 1927, 1965 and 1967 and in the Lister Conference in Edinburgh in February 2012, participants on the programmes were primarily from the medical profession, particularly surgeons. The Lister commemorative conference in London over three days in March 2012 followed in the same tradition of using Lister to assess current issues, but it was unusual in the breadth of issues and range of backgrounds of participants. It demonstrated that Lister's precise position in the history of surgery is still contentious, but his importance as an iconic figure in the history of the medical profession is secure. It is only 14 years until the bicentenary of his birth on 5 April 2027, and it would be a good bet that there will be a commemoration—keep the date free.

## Notes

1 Geoffrey Cubitt, *History and memory* (Manchester University Press, 2007); Pnina G. Abir-Am, ‘Introduction’, in ‘Commemorative practices in science: historical perspectives on the politics of collective memory’ (ed. P. G. Abir-Am and C. A. Elliott), *Osiris* **14**, 1–33 (1999).

2 P. G. Abir-Am and C. A. Elliott (eds), ‘Commemorative practices in science: historical perspectives on the politics of collective memory’, *Osiris* **14** (1999).

3 L. Jordanova, ‘Presidential address: remembrance of science past’, *Br. J. Hist. Sci.* **33**, 387–406 (2000); J. Browne, ‘Presidential address: commemorating Darwin’, *Br. J. Hist. Sci.* **38**, 251–274 (2005); Frank A. J. L. James, ‘Presidential address: the Janus face of modernity: Michael Faraday in the twentieth century’, *Br. J. Hist. Sci.* **41**, 477–516 (2008). Christine MacLeod and Jennifer Tann, ‘ From engineer to scientist: reinventing invention in the Watt and Faraday centenaries, 1919–31’, *Br. J. Hist. Sci.* **40**, 389–411 (2007).

4 See M. Anne Crowther, ‘Lister at home and abroad: a continuing legacy’, *Notes Rec. R. Soc.* **67** (this issue) (http://dx.doi.org/rsnr.2013.0031).

5 This argument is set out fully in A. Crowther and M. Dupree, *Medical lives in the age of surgical revolution* (Cambridge University Press, 2007).

6 These were not the only posthumous Lister commemorations. For example, Lister is unique in having memorial plaques at both King's College, London, and University College London put up after his death. The King's memorial came first in 1914, UCL's in 1919.

7 ‘Lord Lister Buried. Impressive service at Westminster Abbey. A distinguished gathering’, *Westminster Gazette*, 17 February 1912; ‘Lord Lister funeral: service in Westminster Abbey’, *The Times*, 17 February 1912, p. 6.

8 *Ibid.*; ‘The late Lord Kelvin: funeral in Westminster Abbey’, *The Times*, 24 December 1907, p. 4; ‘Lord Lister and Westminster Abbey’, *The Times*, 17 February 1912, p. 9.

9 *Glasgow Herald* (hereafter *GH*), 21 February 1912, p. 8g; 22 February 1912, p. 8f; 27 December 1912, p. 4e.

10 Glasgow City Archive (hereafter GCA), ‘Private meeting, convened by the Lord Provost, in connection with a proposed memorial in Glasgow to the late Lord Lister’, G4/2/1 Minute book—Lister Memorial Fund, 27 February 1912; *GH*, 28 February 1912, p. 8.

11 Minutes of Meeting held in Glasgow on 11 March 1912, reported 14 June 1912, Royal Society Archives (hereafter RSA), CMB/13 Lister Memorial Committee, p. 14.

12 Meeting of the Provisional Committee, RSA, CMB/13, 13 May 1912. William Watson Cheyne (1852–1932), Professor of Clinical Surgery, King's College, London, had been Lister's student, house-surgeon and assistant; Sir John Rose Bradford (1863–1935) was a physician and physiologist who ‘had a gift for organisation’.

13 RSA, CMB/13, 13 and 20 May 1912.

14 RSA, CMB/13, 14 June 1912. The Lord Provost of Glasgow met individually with the Secretary of the Royal Society on 13 May, and a deputation from the Glasgow Committee met with the Provisional Committee in London on 14 June.

15 Minutes of the Meetings of the General Committee and Executive Committee, 22 July 1912, RSA, CMB/13. *GH*, 23 July 1912, p. 7b.

16 Minutes of the Executive Committee, RSA, CMB/13, 4 October 1912.

17 Minutes of the General Committee, RSA, CMB/13, 22 July 1912; Minutes of the Executive Committee, 22 July, 4 and 22 October 1912.

18 Minutes of the Executive and General Committees. RSA, CMB/13, 22 October 1912.

19 Minutes of the Executive Committee, RSA, CMB/13, 29 October 1912. *GH*, 24 October 1912, p. 12g.

20 Minutes of the Executive Committee, RSA, CMB/13, 26 February 1913.

21 Minutes of the Executive Committee, RSA, CMB/13, 25 June 1913.

22 Minutes of the Executive Committee, RSA, CMB/13, 6 July 1914.

23 Comparison with recent prices (2011) is calculated by using http://www.measuringworth.com/ukcompare/ (accessed 12 January 2013). Minutes of the Implementation Sub-Committee, RSA, CMB/13, 24 July 1914.

24 RSA, CMB/13, 25 June 1913, 6 and 24 July 1914.

25 Minutes of the Executive Committee, RSA, CMB/13, 12 March 1920.

26 Minutes of the Executive Committee, RSA, CMB/13, 12 and 26 March, 30 April and 18 June 1920.

27 Minutes of the General and Executive Committees, RSA, CMB/13, 19 July 1920; Minutes of the Executive Committee, 26 November 1921.

28 Minutes of the Executive Committee, RSA, CMB/13, 26 March, 30 April and 18 June 1920.

29 Minutes of the Executive Committee, RSA, CMB/13, 19 July 1920, 26 November 1921, 30 October 1922 and 17 December 1923.

30 Minutes of the Executive Committee, RSA, CMB/13, 17 December 1923, 10 January and 18 February 1924.

31 Minutes of the General Meeting, RSA, CMB/13, 22 July 1912.

32 For a vivid account of the importance of World War I to changing attitudes to mourning in Britain, see, for example, D. N. Cannadine, ‘War and death, grief and mourning in modern Britain’, in *Mirrors of mortality: studies in the social history of death* (ed. J. Whaley), pp. 187–242 (Europa, London, 1981).

33 MacLeod and Tann, *op. cit.* (note 3), p. 400.

34 Meeting of Delegates, RSA, CMB/13, 30 April 1926.

35 Minutes of the Lister Executive Committee, RSA, CMB/13, 6 July 1926.

36 *Br. Med. J.*, 9 April 1927, pp. 683 and 695; 30 July 1927, pp. 185–187.

37 A Logan Turner (ed.), *Joseph Lister: centenary volume 1827–1927 for the Lister Centenary Committee of the British Medical Association* (Oliver & Boyd, Edinburgh, 1927).

38 For insight into the origins of centenary celebrations, see R. Quinault, ‘The cult of the centenary, c.1784–1914’, *Hist. Res*. **71**, 303–323 (1998).

39 Abir-Am, *op. cit.* (note 1), pp. 11–12.

40 For the stamps, see, for example, http://www.bfdc.co.uk/1965/antiseptic_surgery/lister_medal.html; Charles Illingworth (ed.), *Wound healing: a symposium based upon the Lister Centenary Scientific meeting held in Glasgow, September 1965* (J. & A. Churchill Ltd, London, 1966), pp. v and viii.

41 Illingworth, *op. cit.* (note 40), p. viii.

42 Lister Centenary Conference April 2nd–6th, 1967 Exhibition Catalogue, Schedule and Programme, The King's College, London (Weston Education Centre Library) (hereafter KCL(WECL)), LC/1, LC/5, LC/6.

43 *Br. Med. J.*, 15 April 1967, p. 172. File of letters concerning Lister celebrations 1966–. ‘Memo re: Programme, 23 March 1967’, KCL(WECL), LC/42.

44 KCL(WECL), LC/6 Programme, p. 9.

45 *GH*, 4 December 1912, p. 9b; 12 December 1912, p. 9b; 27 December 1912, p. 4e–g.

46 *GH*, 28 November 1912, p. 8f.

47 Glasgow Royal Infirmary (GRI) Minute Book, 1912: 7 March 1912, National Health Service Greater Glasgow and Clyde Archives (hereafter NHSGGCA) HB14/1/27; J. D. Hedderwick to P. Rintoul, 11 March and 21 March 1912.

48 For an account of the reconstruction of the GRI, see J. Jenkinson, M. Moss and I. Russell, *The Royal: the history of the Glasgow Royal Infirmary, 1794–1994* (Glasgow Royal Infirmary Bicentenary Committee, Glasgow, 1994).

49 *Lister and the Lister Ward in the Royal Infirmary of Glasgow: a Centenary Contribution* (hereafter *Lister Ward*) (Jackson, Wylie & Co., Glasgow, 1927), pp. 84–85.

50 NHSGGCA HB14/29 Minute Book 1914, p. 54, April 1914; p. 72, 6 May 1914; *Lister Ward*, pp. 117–119.

51 *Lister Ward*, p. 85.

52 NHSGGCA HB14/29 Minute Book 1914, pp. 87–100, 6 May 1914; 15 May 1914; 2 June 1914; 10 June 1914. ‘The Lister Ward—plea for retention’, *GH*, 3 March 1922.

53 Jenkinson *et al.*, *op. cit.* (note 48), pp. 170–171.

54 NHSGGCA HB14/36 Minute Book 1921, pp. 92–93.

55 *Ibid.*, 18 October 1921.

56 Ibid.

57 *Lister Ward*, pp. 85–86. ‘Appeal for retention’, *GH*, 3 February 1922; ‘The Lister Ward—plea for retention’, 3 March 1922; ‘Royal Infirmary—The Lister Ward’, 18 April 1922; ‘The Lister Ward’, 21 July 1922.

58 *GH*, 18 April 1922.

59 *GH*, 15 January 1923; ‘Vandalism at Glasgow’, *The Times*, 3 October 1923; ‘Letter from Lord Blytheswood’, *The Times*, 11 October 1923; ‘Vandalism at Glasgow’, *GH*, 12 October 1923. ‘Appendix II—A Humble Plea … by James A. Morris published Sept 1923’, in *Lister Ward*, pp. 112–121.

60 ‘Lister Ward Protest by French Academy’, *GH*, 13 October 1923; ‘The Lister Ward—public appeals for retention’, *GH*, 3 November 1923; ‘Appeals for retention’, *GH*, 1 December 1923. *Lister Ward*, pp. 86–88; ‘Appendix I—list of public appeals’, *Lister Ward*, pp. 105–111.

61 *GH*, 4 December 1923; ‘Special Meeting of the Managers’, NHSGGCA HB14/37 Minute Book 1923–4, pp. 169–171, 3 December 1923.

62 ‘Works Committee’, NHSGGCA HB14/37 Minute Book 1923–4, 28 January 1924; ‘The Lister Ward—further plea by James A Morris’, *GH*, 7 February 1924.

63 *The Times*, 5 March 1924, p. 7.

64 *GH*, 16 May 1924.

65 A fuller account of the controversy will appear in a forthcoming article by M. Dupree: ‘Commemoration and controversy: the case of the Lister Ward’.

66 C. J. S. Thompson to Dr Maxton Thom [Superintendent, Glasgow Royal Infirmary], Wellcome Archives (WA)/HMM/EX/A.3, 16 January 1924; Dr Thom to C. J. S. Thompson, *loc. cit.*, 17 January 1924; C. J. S. Thompson to Messrs P. & W. Anderson [building contractors], *loc. cit.*, 18 January 1924; C. J. S. Thompson to Messrs P. & W. Anderson, *loc. cit.*, 29 January 1924.

67 For a full account of Wellcome and his collection, see Frances Larson, *An infinity of things: how Sir Henry Wellcome collected the world* (Oxford University Press, 2009).

68 L. W. G. Malcolm, ‘The Lister Exhibition in London’, in *Lister Ward*, p. 95.

69 *Ibid.*, pp. 171 and 209.

70 C. J. S. Thompson to Dr Thom, WA/HMM/EX/A.3, 2 April 1924; Dr Thom to C. J. S. Thompson, *loc. cit.*, 7 May 1924; C. J. S. Thomson to Dr Thom, *loc. cit.*, 7 June 1924.

71 WA/HMM/RP/MAL/1, Report/MM8 25/v/1926.

72 Malcolm to Henry Wellcome, WA/HMM/RP/MAL/1, 25 May 1926.

73 WA/HMM/RP/MAL/1, Report/MM9 8/vi/1926.

74 Malcolm to Wellcome, WA/HMM/RP/MAL/1, 22 June 1926.

75 Circular letter from Johnston Saint, WA/HMM/EX/A.4, 28 September 1927.

76 See Crowther, *op. cit.* (note 4).

